# The associations of emotion regulation, self-compassion, and perceived lifestyle discrepancy with breast cancer survivors’ healthy lifestyle maintenance

**DOI:** 10.1007/s11764-024-01656-6

**Published:** 2024-08-24

**Authors:** Tal Jean Ben-Artzi, Svetlana Baziliansky, Miri Cohen

**Affiliations:** School of Social Work, University of Haifa, Carmel Mountain, Haifa, 3103301 Israel

**Keywords:** Breast cancer survivors, Healthy lifestyle, Physical activity, Healthy diet, Self-compassion, Emotion regulation, Perceived lifestyle discrepancy

## Abstract

**Purpose:**

Unhealthy lifestyle increases the risk of comorbidities, reduced quality of life, and cancer recurrence among breast cancer survivors. It is important to identify emotional and cognitive factors that may affect the maintenance of a healthy lifestyle over time. This study examined the associations of perceived lifestyle discrepancy, self-compassion, and emotional distress with the maintenance of a healthy lifestyle among breast cancer survivors and the mediating role of emotion regulation patterns (cognitive reappraisal and expressive suppression) in these associations.

**Methods:**

A total of 145 female breast cancer survivors aged 31–77 completed self-reports on healthy lifestyle maintenance, perceived lifestyle discrepancy, self-compassion, emotional distress, and emotion regulation patterns. Structural equation modeling was used to analyze the data.

**Results:**

Mean physical activity and healthy diet maintenance scores were moderate. The structural equation modeling analysis showed good fit indicators (χ2 = 4.21, df = 10, p = .94; χ2/df = 0.42; NFI = .98; TLI = 1.09; CFI = 1.00; RMSEA = .00, 95% CI (.00, .02)). Lower perceived lifestyle discrepancy was directly associated with higher physical activity (β = −.34, p < .01) and healthy diet (β =−.39, p < .01). Cognitive reappraisal was associated with higher physical activity (*β* = .19, *p* < .01), and expressive suppression was associated with lower physical activity (*β* = −.19, *p* < .01), and both mediated the association between self-compassion and physical activity.

**Conclusions:**

The mediated associations reported in this study indicate that psychosocial factors, especially self-compassion, perceived lifestyle discrepancy, and emotional regulation patterns, are relevant to healthy lifestyle maintenance among breast cancer survivors, because solely providing healthy lifestyle recommendations does not motivate individuals to adhere to them.

**Implications for Cancer Survivors:**

Short-term structured psychosocial interventions designed to reduce perceived health discrepancy and strengthen self-compassion should be implemented and their effect on lifestyle should be further evaluated.

## Introduction

Breast cancer is the most common cancer among women worldwide [1]. In Israel, the occurrence rate is higher than in most countries [2]. The overall 5-year survival rate for women diagnosed with breast cancer is between 85 and 90% in high-income countries [1] and 89% in Israel [2].

In recent years, research and clinical evidence have accumulated showing that cancer survivors experience various long-term physical and mental symptoms, such as depression, anxiety, fatigue, and cognitive difficulties [3]. About 90% of breast cancer survivors experience varying levels of these symptoms 1 year after diagnosis, and about 30% continue to experience these symptoms many years posttreatment [3]. These difficulties often impair the personal, family, marital, and social functioning of survivors; reduce their quality of life [3]; and may even affect survival time [4]. Moreover, compared to the general population, breast cancer survivors are at higher risk of developing chronic conditions, such as diabetes, cardiovascular diseases, and osteoporosis, and cancer-related and all-cause mortality [5].

An unhealthy lifestyle, including an unbalanced diet, low physical activity, and overweight, among breast cancer survivors was found to be an established risk factor for development of secondary cancers and other chronic conditions [5], all-cause and cancer-related mortality, increased long-term symptoms, and reduced quality of life [5, 6]. Moreover, a healthy lifestyle has been suggested as a key factor that can improve long-term health and survival among cancer survivors [5, 7, 8]. For example, a recent meta-analysis of 49 studies reported a 23% reduction in overall mortality among breast cancer survivors who embraced a healthy diet [7].

Despite the proven beneficial effects of a healthy lifestyle for cancer survivors [4], maintenance of recommended lifestyle guidelines is low among this group [9, 10]. Low healthy lifestyle maintenance has also been well documented in the general population [11]. Most studies of the general population focused on environmental barriers (e.g., financial, accessibility, social support) [12, 13]. Also, existing theoretical models of behavior change focus on the role of cognitive barriers (e.g., norms, perceived benefits of change, perceived control, perceived self-efficacy) [14]. In contrast, scholars have suggested that human behaviors, including health behaviors, are driven to a large extent by psychological and emotional factors [14], but only a few studies have examined the effect of these factors on health behaviors [14].

As for cancer survivors, beside recent systematic reviews that identified demographic, physical functioning, and symptom-related factors [9, 15], only a few studies have examined psychosocial factors. The main psychosocial factors related to better engagement in a healthy lifestyle were perceptions and attitudes (exercise self-efficacy, perceived behavioral control, intention) or psychological well-being and lower emotional distress [16–18]. Another group of studies examined intrinsic motives for participation in physical and psychological interventions, mainly to improve their physical and psychological well-being [19]. Nevertheless, more studies are needed to understand the role of psychosocial processes that affect health behaviors among cancer survivors [4, 20].

A main theoretical model for examining factors affecting individuals’ behaviors and emotions in stressful encounters is the model of stress and coping [22]. The model suggests that coping resources, cognitive appraisals of the situation, and coping patterns significantly affect emotional reactions and behaviors related to a stressful situation [22], whereas coping patterns such as emotion regulation strategies mediate these relations.

Personal coping resources are defined as individual characteristics contributing to effective processes of coping with stressful situations [22]. Self-compassion is a significant personal resource—it is self-kindness and the ability to acknowledge and accept suffering and pain, rather than ignoring or criticizing personal negative feelings [20, 22]. It is characterized by three main components: common humanity (recognizing that suffering or failures are part of the human experience), self-kindness (being kind to and understanding of oneself), and a balanced approach (accepting negative emotions so they are neither suppressed nor exaggerated) [19, 22]. It also entails compassion, kindness, and care of the body [24]. Self-compassion was identified in several studies as an important resource for coping with cancer [19] related to lower psychological distress [23, 24]. A few studies noted the association between self-compassion and healthier lifestyle behaviors [25], but no research has examined associations between self-compassion and healthy lifestyle among cancer survivors.

Cognitive appraisals of stressful situations play a significant role in the experience of stress [22]. A factor that may affect the maintenance of a healthy lifestyle is the perceived discrepancy between breast cancer survivors’ desire to improve their health and actual maintenance of physical activity and nutritional recommendations [20]. The effect of this gap on actual behavior may be explained by the theory of cognitive dissonance [26], which refers to the state of discomfort created by holding two or more pieces of knowledge that are connected yet inconsistent with each other. For example, conflicting beliefs, perceptions, attitudes, or motivations could lead to cognitive dissonance [26]. Researchers have acknowledged the central role of cognitive dissonance (or similar variables) in health-related decisions, such as meat consumption, dietary restraint, and abstinence-promoting health messages [27]. Dissonance can promote behavioral change or increase avoidance of health-related information [29, 30]. Nevertheless, most studies in this domain involved the general population and not cancer survivors [28].

Studies have pointed to the central role of emotional regulation in individuals’ behaviors [31]. Emotion regulation has been conceptualized as coping patterns that modulate how individuals experience and express emotions, both consciously and unconsciously [31]. Two main emotion regulation patterns are expressive suppression and cognitive reappraisal [31]. Expressive suppression has been conceptualized as a conscious process whereby individuals inhibit their emotion-expressive behavior [31]. Cognitive reappraisal involves positive interpretations of or perspectives on stressful circumstances to reduce psychological distress [31]. Emotion regulation patterns have been frequently examined in the context of coping with cancer [32], but not in the context of healthy lifestyle maintenance.

**Fig. 1:**
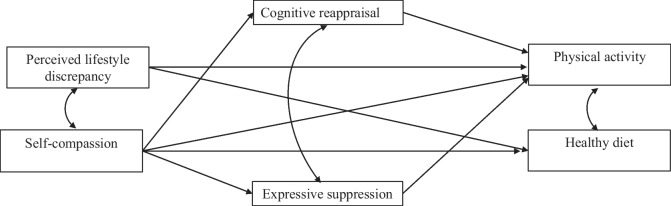
Suggested study model

Research on psychological mechanisms that may affect the maintenance of a healthy lifestyle is scarce. Therefore, this study aimed to expand our understanding of the role of psychological factors in this regard, especially factors not yet examined. Specifically, based on the model of coping with stress [21], we aimed to examine the associations of self-compassion, perceived discrepancy, and emotion regulation patterns with healthy lifestyle maintenance (physical activity and healthy diet) among breast cancer survivors, along with the mediating role of emotion regulation patterns between self-compassion and perceived discrepancy and healthy lifestyle. Our hypotheses were that self-compassion, perceived discrepancy, and emotion regulation patterns would be directly associated with healthy lifestyle maintenance and that emotion regulation patterns would mediate the associations between self-compassion and perceived discrepancy and healthy lifestyle (Fig. 1).

## Methods

### Participants and procedure

This was a cross-sectional study. The study was approved by the ethical board of [440/22]. Data were collected from March to May 2023. Participants were 145 female breast cancer survivors aged 31–77. Inclusion criteria included completion of treatment for breast cancer (surgery, chemotherapy, radiotherapy, immune, or biological treatment) at least 1 year prior to the study. Exclusion criteria were current recurrence of cancer and age younger than 30. Participants were recruited through social media groups and webpages designed for breast cancer survivors (e.g., Facebook, cancer survivor groups and organizations) and the snowball method. Potential participants received a link to an online document presenting the study, the inclusion and exclusion criteria, and an online consent form. Then, they filled in the questionnaire online using Qualtrics software. In a separate Qualtrics questionnaire, they provided their email address and received a digital coupon with a $22 value as a token of appreciation.

Although 147 participants initially responded, two participants were excluded from the study due to incomplete questionnaires. These participants resigned immediately after confirming the inclusion criteria, and all other questions were mandatory; therefore, we had 100% responsiveness among the 145 participants. The needed sample size was determined using Cohen’s formula for power analysis [33]: For a multiple regression analysis with eight predictors (four study variables and four confounders to control), when a medium effect size is desired at power = .80 and alpha = .05, 107 participants are needed. A priori sample size calculation for structural equation modeling analysis with eight predictors, a small effect size, and a power of .80 indicated that the minimum sample size is 100 [34].

### Measures

#### Demographic and health-related history

Demographic and cancer-related details included age, employment status, marital status, number of children, and education. In addition, the participants provided information on breast cancer history, including year of cancer diagnosis, cancer stage at diagnosis, type of treatments, chronic health conditions, and cancer-related symptoms, examined using the EORTC QLQ-C30 [35]. Participants reported their height and weight, enabling body mass index (BMI) to be calculated.

#### Lifestyle assessment

Physical activity was examined using the General Practice Physical Activity Questionnaire [36]. This measure consists of two parts: (a) the amount of physical activity involved in work, described with five levels of intensity (e.g., “My work involves vigorous physical activity including handling of very heavy objects”), with participants asked to choose one category that describes them the most; and (b) five items referencing physical exercise (intensive swimming, jogging, aerobic exercises, cycling, housework, and gardening). Participants indicated the number of hours per week they spend engaged in physical activity: more than 3, 1–3, less than 1, or none. Two items, house and garden work, were excluded from the data analysis due to their low validity [36]. The group that developed this tool suggested excluding these items due to overlap with the other items [36]. The sum scores of the two scales were categorized into four categories—(a) inactive, (b) moderately inactive, (c) moderately active, and (d) active—as suggested [36].

Healthy diet was examined by the Israeli Mediterranean Diet Screener [37]. We used eight items examining the number of servings consumed by participants from different food groups per day (except for alcohol, which was examined as servings per week). Depending on the number of servings indicated, participants received either 1 or 0 points [38]. A sum score was calculated, with a possible range of 0–8 points; higher scores indicated a healthier diet. A cutoff score for healthy eating was not provided by the authors of this scale [37].

#### Perceived lifestyle discrepancy

Perceived lifestyle discrepancy was assessed with an item developed for the current study due to the lack of lifestyle discrepancy questionnaires appropriate for the study. The item was as follows: “How aligned is your lifestyle with the recommended lifestyle?” Participants were asked to rate their level of alignment on a 5-point Likert scale from 0 (*very low discrepancy*) to 4 (*very high discrepancy*).

#### Emotion regulation

Emotion regulation was measured with the 10-item Emotional Regulation Questionnaire [31], measuring an individual’s tendency to use cognitive reappraisal (six items, e.g., “I control my emotions by changing the way I think about the situation I’m in”) or expressive suppression to regulate negative emotions (four items, e.g., “I control my emotions by not expressing them”). The items were scored on a 7-point scale from 0 (*do not agree at all*) to 6 (*agree always*). Mean scores were calculated, with higher scores indicating higher levels of expressive suppression or cognitive reappraisal. The internal consistency of the Hebrew version was *α* = .95 for expressive suppression and *α* = .89 for cognitive reappraisal [32]. In the present study, internal consistency was *α* = .81 for expressive suppression and *α* = .90 for cognitive reappraisal.

#### Self-compassion

Self-compassion was examined using the Self-Compassion Scale Short Form [39]. The 12-item questionnaire includes six self-compassion categories: kindness toward oneself, self-judgment, common humanity, loneliness, mindfulness, and overidentification. Participants were asked to rate how much they agree with the phrases on a 5-point scale from 1 (*almost never*) to 5 (*almost always*). The internal consistency of the initial questionnaire was *α* = .86 [39]. The questionnaire was translated into Hebrew, and its internal consistency was *α* = .86 [32]. In the current study, internal consistency was *α* = .86.

#### Emotional distress

Emotional distress was examined using the depression and anxiety subscales of the Brief Symptom Inventory-18 [40]. Participants were asked to rate their feelings during the previous 7 days on a 5-point scale from 0 (*not at all*) to 4 (*extremely*). Mean scores for the depression and anxiety subscales were calculated, with higher scores indicating higher distress. Internal consistency was *α* = .86 for the Hebrew version [32] and *α* = .92 in the current study.

### Data analysis

SPSS 27 and AMOS 27 were used for data analysis. Descriptive statistics and Pearson correlations were used to explore the associations among study variables and between the study variables and demographic variables. The research model was processed using a structural equation modeling path analysis with IBM AMOS 27.0, with the goal of assessing direct and indirect pathways from resilience to PTSS. Perceived lifestyle discrepancy and self-compassion served as independent variables; physical activity and healthy diet were the independent variables, and both emotion regulation patterns served as mediators. In addition, the associations between potential confounders and the two dependent variables (healthy diet and physical activity) were examined to determine what confounders should be added to the model. Cancer-related symptoms, age, and number of children were associated with either physical activity or healthy diet and thus entered in the model. Model fit was assessed using the following indexes: chi-square and normed chi-square tests to assess the model’s overall fit and parsimony; comparative fit index (CFI) to examine the discrepancy between the data and the hypothesized model while adjusting for issues of sample size; Tucker-Lewis Index (TLI) and normed fit index (NFI), which measure of goodness of fit and are not affected by the number of parameters in the model; and root-mean-square error of approximation (RMSEA) and its confidence interval (CI), which measure the discrepancy per degree of freedom and indicate the model’s absolute fit. In addition, indirect effects were evaluated using a bootstrapping method (5000 bootstrap samples) and 95% bias-corrected CIs to evaluate the statistical significance of indirect paths.

## Results

### Demographic and cancer-related characteristics

The participants’ demographic details are presented in Table 1. Their average age was 53 (*SD* = 53.84) years. Most participants were married (65.5%) and employed (75%). Participants were on average about 5 (*SD* = 4.06) years postdiagnosis. About a third (36.5%) were diagnosed with tumor in situ or stage I, another third (35.9%) at stage II, about 22.8% at stage III, and less than 5% at stage IV. Nearly 5% of the participants underwent surgery only; about 55% of the participants received both chemotherapy and radiation therapy, and another 30% had undergone immunotherapy or biological therapy, mostly in addition to other therapies. About half of the participants received hormonal treatment. Fatigue was the most intense symptom (*M* = 2.81, *SD* = 0.84), followed by sleep disturbances (*M* = 2.63, *SD* = 0.90), weakness (*M* = 2.39, *SD* = 0.90), and pain (*M* = 2.37, *SD* = 0.91). On average, the mean number of chronic health conditions reported was 2.84 (*SD* = 3.43); the most frequent diseases were cardiovascular (35.2%), followed by lung (31.7%), endocrine (31.7%), gastrointestinal (31.7%), and kidney (30.3%) diseases; and 37.9% reported no chronic diseases. Participants reported a medium level of cancer-related symptoms.

**Table 1:** Demographic and health details of participants

	*M* or *n*	*SD* or %	Range
Age, years (*M*, *SD*)	53.84	9.76	31–77
Education, years (*M*, *SD*)	15.56	2.69	11–28
Employed (*n*, %)	108	75.0	
Married or partnered (*n*, %)	95	65.5	
Number of children (*M*, *SD*)	2.92	1.56	0–10
Cancer stage (*n*, %)			
In situ	18	12.4	
Stage I	35	24.1	
Stage II	52	35.9	
Stage III	33	22.8	
Stage IV	7	4.8	
Cancer-related symptoms (*n*, %)	2.33	.63	1.00–3.78
Treatment^a^ (*n*, %)			
Chemotherapy	91	62.8	
Radiation	123	84.8	
Hormonal or endocrine	79	54.5	
Immunotherapy	4	2.8	
Biological	39	26.9	
Surgery only	7	4.8	
Time since diagnosis, years (*M*, *SD*)	5.07	4.06	1–23
Number of chronic conditions (*M*, *SD*)	2.84	3.43	0–9
BMI (*M*, *SD*)	25.92	4.74	16.56–40.40

^a^Most participants received more than one treatment

### Means and standard deviations of study variables

Table 2 shows the means, standard deviations, ranges, and associations of the study variables. Mean scores of physical activity and healthy diet were moderate. In addition, 22.8% of participants were inactive, 48.2% were low or moderately active, and only 29% were in the active category, thus meeting the recommended level of activity. Also, nearly 41% of the participants indicated that their work was mainly sedentary. In relation to the recommended Mediterranean diet, 66% of the participants indicated using olive oil as their main oil source and 42% reported consuming at least five servings of vegetables and fruits a day. Slightly more than half of the participants indicated eating one or no servings of whole grains a day. The participants’ mean BMI score was 25.92 (*SD* = 4.74), which slightly exceeds the recommended BMI range [41].

**Table 2:** Means, standard deviations, ranges, and Pearson correlations for study variables

	*M* (*SD*)	Actual range	1	2	3	4	5	6
1. Healthy diet	4.05 (1.19)	4–6						
2. Physical activity	2.64 (1.13)	1–4	.33***					
3. Self-compassion	3.14 (0.69)	1.25–4.83	.14	.25**				
4. Perceived discrepancy	3.06 (0.98)	1–5	−.40***	−.40***	−.29***			
5. Cognitive reappraisal	4.16 (1.30)	1–7	−.01	.15	.39**	−.05		
6. Expressive suppression	2.72 (1.21)	1.00–5.75	−.09	−.21*	−.30***	.20*	.16	
7. Emotional distress	1.95 (0.68)	1.00–3.83	.06	−.10	−.58***	.22**	−.31***	30**

*p < .05; **p < .01; ***p < .001

The mean scores of the independent variables, self-compassion and perceived discrepancy, were close to the middle of their possible ranges (*M* = 3.14, *SD* = 0.69 and *M* = 3.06, *SD* = 0.98, respectively). Cognitive reappraisal was moderate (*M* = 4.16, *SD* = 1.30), and regulating emotions with expressive suppression was relatively low (*M* = 2.72, *SD* = 1.21). Emotional distress was relatively low (*M* = 1.95, *SD* = 0.68).

### Associations between study variables

Pearson correlation coefficients were calculated (Table 2). Maintaining a healthy diet and engaging in physical activity were negatively associated with perceived discrepancy (*r* = −.40, *p* = .01). In addition, physical activity had a weak positive correlation with self-compassion (*r* = .25, *p* < .01) and a weak negative association with expressive suppression (*r* = −.21, *p* < .05), whereas healthy diet was not associated with other variables except lifestyle discrepancy. No lifestyle variables were associated with emotional distress, and thus, we excluded the emotional distress variable from additional analysis.

### Study model

Self-compassion and perceived discrepancy were the independent variables in the model; cognitive reappraisal and expressive suppression were the mediators, and healthy diet and physical activity were the outcome variables. Health-related quality of life, age, and number of children were entered as confounders. The final model showed good fit: χ2 = 4.21, df = 10, p = .94; χ2/df = 0.42; NFI = .98; TLI = 1.09; CFI = 1.00; RMSEA = .00, 95% CI (.00, .02).

**Fig. 2:**
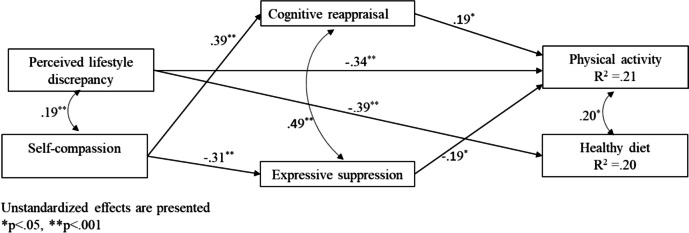
Direct and indirect associations between study variables

The model revealed several direct effects (Fig. 2): negative associations of perceived discrepancy with physical activity (*B* = −.34, *p* < .001) and healthy diet (*B* = −.39, *p* < .001), along with associations of self-compassion with both emotional regulation patterns—positively associated with cognitive reappraisal (*B* = .39, *p* < .001) and negatively associated with expressive suppression (*B* = −.31, *p* < .001). Associations were also found between emotion regulation patterns and physical activity—positively associated with cognitive reappraisal (*B* = .19, *p* < .05) and negatively associated with expressive suppression (*B* = −.39, *p* < .001)—but not with healthy diet. Bootstrapping analysis showed that self-compassion was associated with physical activity via cognitive reappraisal and expressive suppression. The higher the self-compassion, the higher the use of cognitive reappraisal and the higher the physical activity (*B* = .13, 95% CI (.04, .22)). The higher the self-compassion, the lower the use of expressive suppression and the higher the physical activity (*B* = −.07, 95% CI (.01, .20)).

## Discussion

This study was conducted to increase knowledge on the associations of psychosocial factors with the maintenance of a healthy lifestyle among breast cancer survivors. The main results show moderate levels of physical activity and healthy diet maintenance. Whereas healthy diet maintenance was associated only with lower perceived discrepancy, higher physical activity was also associated with higher self-compassion, lower use of expressive suppression, and higher use of cognitive reappraisal. Moreover, self-compassion was associated with physical activity via the mediation of cognitive reappraisal and expressive suppression.

Although the average level of activity in the present study was moderate, the reported levels of activity varied widely, with about 70% of participants under the suggested activity level, in line with previous reports [28]. Although previous studies used various measures of activity, similar rates of insufficient activity were reported [10, 42]. For example, in Busen and colleagues’ study [42], nearly 60% of the participants did not meet recreational physical activity guidelines. In another study, 41.6% of survivors reported not partaking in any exercise or engaging in low levels of physical activity [10]. Similarly, 41% of participants in the current study indicated that their work was mainly sedentary.

Regarding healthy diet, the mean score showed a moderate level of adherence to daily diet guidelines, in accordance with previous reports [37]. Although it is difficult to compare studies due to different measures [7], low scores for healthy diet maintenance have been generally observed among breast cancer survivors [4, 43]. Regarding diet elements, the figures in the present study were lower than the World Cancer Research Fund/American Institute for Cancer recommendations [8]. In the present study, 57.9% of participants indicated eating four or fewer servings of fruits and vegetables a day, whereas the recommendation is at least five daily servings of nonstarchy vegetables and fruits [8]. High intake of fruits and vegetables is a primary element of a healthy diet and related to better prognostic factors among cancer survivors [8]. However, the present finding indicates higher consumption of fruits and vegetables compared to former studies, for example, in Karavasiloglou and associates’ study [44], cancer survivors indicated eating 2.4 servings of vegetables and 1.0 servings of fruit a day. Regarding whole-grain consumption, in the current study, 53% of participants indicated eating one or no servings of whole grains a day, which is lower than the recommendation to eat whole grains in most meals [8]. Various studies have noted the importance of whole-grain consumption. For example, a meta-analysis found that three daily servings of whole grains lower the risk of mortality from cardiovascular diseases by 25%, all-cause mortality by 17%, and total cancer mortality by 10% [45]. Although previous studies suggested various environmental, demographic, and cancer-related factors and cognitive perceptions and attitudes [9, 15–18], the present results offer a theoretically based model [21] of associations between coping resources and strategies and healthy lifestyle maintenance, as further discussed.

Following the coping theory [21], healthy lifestyle discrepancy is a cognitive perception that may influence an individual’s emotions and behaviors. Accordingly, this study indicated that the higher the discrepancy between suggested and actual healthy lifestyle maintenance among participants, the less healthy their diet was and the less they engaged in physical activity, an association which can be explained in several ways. This finding may suggest that being aware of this discrepancy may evoke uncomfortable feeling of low self-efficacy and low ability to follow recommendations, which may decrease motivation to engage in healthy behaviors [19]. This explanation accords with the theory of cognitive dissonance [26]. Higher dissonance is often related to avoidance of health-related information [30]. Second, survivors may be less motivated to change their health behaviors [19]. Third, nowadays, cancer survivors can access many different and at times contradictory lifestyle recommendations. Survivors who perceive a discrepancy between their lifestyle and the recommended lifestyle might feel they are always “wrong” due to confusing recommendations, which may lower their motivation to practice a healthy diet. An additional explanation of this finding aligns with previous studies [46] indicating that solely holding information about health behaviors is not enough to motivate healthy lifestyle maintenance. For example, studies found that understanding the importance of routine screening for early cancer detection does not ensure involvement in screening [46], although other studies with populations not affected by cancer found higher cognitive dissonance and increased engagement in a healthy lifestyle [29]. Nevertheless, the association between health discrepancy and healthy lifestyle can be explained in the other direction—that is, cancer survivors who are aware of their unhealthy eating might perceive higher discrepancy.

The study model showed that lifestyle discrepancy was only directly associated with the healthy lifestyle components, and these associations were not mediated via emotion regulation patterns. This finding contrasts with the coping theory [21], which posits that coping mediates the effect of cognitive perceptions on emotional and behavioral outcomes. However, perceived discrepancy had not been previously examined in relation to emotion regulation patterns, other coping strategies, or healthy lifestyle among cancer survivors.

Self-compassion, a major personal resource related to efficient coping with long-term stressors [22, 47], has been seldom studied in relation to health behaviors among cancer survivors [19]. Our study supports previous findings in various populations that self-compassion was positively associated with engagement in health behaviors, including physical activity [25, 47], although others found no associations [48]. This association was previously explained by the positive effect of self-compassion on motivation, overcoming barriers to maintaining a physical activity regimen [49], and efficient coping patterns [22].

In accordance with some previous findings, the present results indicate that cognitive reappraisal was associated with better physical activity [50], whereas expressive suppression usually was related to worse outcomes [51]. This is the first study to the best of our knowledge to show the mediational effect of emotion regulation patterns between self-compassion and physical activity among cancer survivors. Our findings suggest that individuals with high self-compassion tend to acknowledge their hardships and accept them; then, they shift their view of the situation to a more positive or manageable one, as former research found [19, 47, 51]. A previous study supported the negative association between self-compassion and expressive suppression [51], suggesting that coping patterns mediate the relationship between personal resources and emotional and behavioral outcomes [21]. Nevertheless, due to the correlational nature of the results, it may be that higher engagement in physical activity improves self-compassion and enables more adaptive coping patterns.

In contrast to the mediational associations between self-compassion and physical activity, self-compassion was not correlated with healthy diet, neither directly nor indirectly via emotion regulation. This contradicts former findings that self-compassion was correlated with better adherence to a healthy lifestyle [25, 47]. However, these studies were conducted in the general population and assessed healthy eating mainly by asking participants to share their subjective perception of their eating habits [25, 47], which may have been biased by social desirability [52].

The lack of an association between emotional distress and healthy lifestyle contradicts previous findings in the general population [53] and among cancer survivors [6], especially given findings of bidirectional influences between lifestyle and emotional states [53]. The lack of associations may be explained by the low emotional distress reported by the participants that may not affect their health behaviors, as previously reported [17].

### Implications for practice

These results have several implications for practice. First, our findings suggest that to improve survivors’ lifestyle, interventions should be tailored to address perceptions of discrepancy and strengthen self-compassion and positive emotion regulation strategies. This accords with previous studies that found providing health information or referring survivors to lifestyle consultations may not be sufficient for healthy lifestyle change or long-term maintenance [46]. We suggest identifying cancer survivors with high discrepancy regarding their health behaviors and initiating discussions on ways to reduce this discrepancy using dissonance-based interventions [54]. Further, we encourage health professionals to explore their sense of perceived discrepancy regarding their own lifestyle and how it may influence lifestyle suggestions for clients.

Psychosocial oncology professionals can help cancer survivors strengthen self-compassion and cognitive reappraisal strategies and discuss how challenging yet important it is to adhere to a healthy lifestyle. These could be accomplished by providing simple yet significant self-compassion practices to manage feelings of being overwhelmed, including increased awareness of self-judgment, feelings of inadequacy, and intolerance of disliked personal characteristics, along with mindfulness and acceptance [19]. To achieve this goal, we suggest that professionals adopt intervention techniques from acceptance and commitment therapy or compassion cultivation training models previously adapted for cancer patients [23].

Several limitations of the study should be noted. A main limitation is the cross-sectional design; therefore, caution is needed in inferring the directionality of the results. In addition, the study collected data online. Therefore, breast cancer survivors who have lower access to the internet or social media might not have been able to participate in the study, which may limit the study’s generalizability to other breast cancer survivors. Another limitation of the data collection method is that participants reported their health- and cancer-related history. Further, because scales measuring cognitive dissonance in relation to healthy lifestyle are lacking, one item was composed to measure perceived lifestyle discrepancy. Further validation of this item is warranted. Due to social desirability, participants may have answered questions in accordance with their desired lifestyle choices rather than actual choices. Nevertheless, this study is among a few to examine psychological variables that may directly and indirectly influence the maintenance of a healthy lifestyle, which is critical for quality of life and survival among breast cancer survivors. Further research is needed regarding factors that affect healthy lifestyle maintenance, such as body compassion [23], and development and validation of a perceived discrepancy questionnaire for cancer survivors. Finally, further examining factors associated with healthy lifestyle among survivors of breast and other cancers with longitudinal or ecological momentary designs is suggested.

## Data Availability

Data will be available upon a reasonable request.
